# Dynamic tuning of FRET in a green fluorescent protein biosensor

**DOI:** 10.1126/sciadv.aaw4988

**Published:** 2019-08-07

**Authors:** Pablo Trigo-Mourino, Thomas Thestrup, Oliver Griesbeck, Christian Griesinger, Stefan Becker

**Affiliations:** 1 Department for NMR-Based Structural Biology, Max Planck Institute for Biophysical Chemistry, Göttingen, Germany.; 2 Structural Elucidation Group, Analytic Enabling Technologies, Merck & Co., 2015 Galloping Hill Road, Kenilworth, NJ 07033, USA.; 3 Max Planck Institute of Neurobiology, Martinsried, Germany.

## Abstract

Förster resonance energy transfer (FRET) between mutants of green fluorescent protein is widely used to monitor protein-protein interactions and as a readout mode in fluorescent biosensors. Despite the fundamental importance of distance and molecular angles of fluorophores to each other, structural details on fluorescent protein FRET have been missing. Here, we report the high-resolution x-ray structure of the fluorescent proteins mCerulean3 and cpVenus within the biosensor Twitch-2B, as they undergo FRET and characterize the dynamics of this biosensor with $B_{0}^{2}$-dependent paramagnetic nuclear magnetic resonance at 900 MHz and 1.1 GHz. These structural data provide the unprecedented opportunity to calculate FRET from the x-ray structure and to compare it to experimental data in solution. We find that interdomain dynamics limits the FRET effect and show that a rigidification of the sensor further enhances FRET.

## Teaser

The dynamic nature of FRET sensor Twitch-2B, as probed by x-ray, NMR, and modeling, reveals key information on how to tune FRET.

## INTRODUCTION

**Fig. 1:**
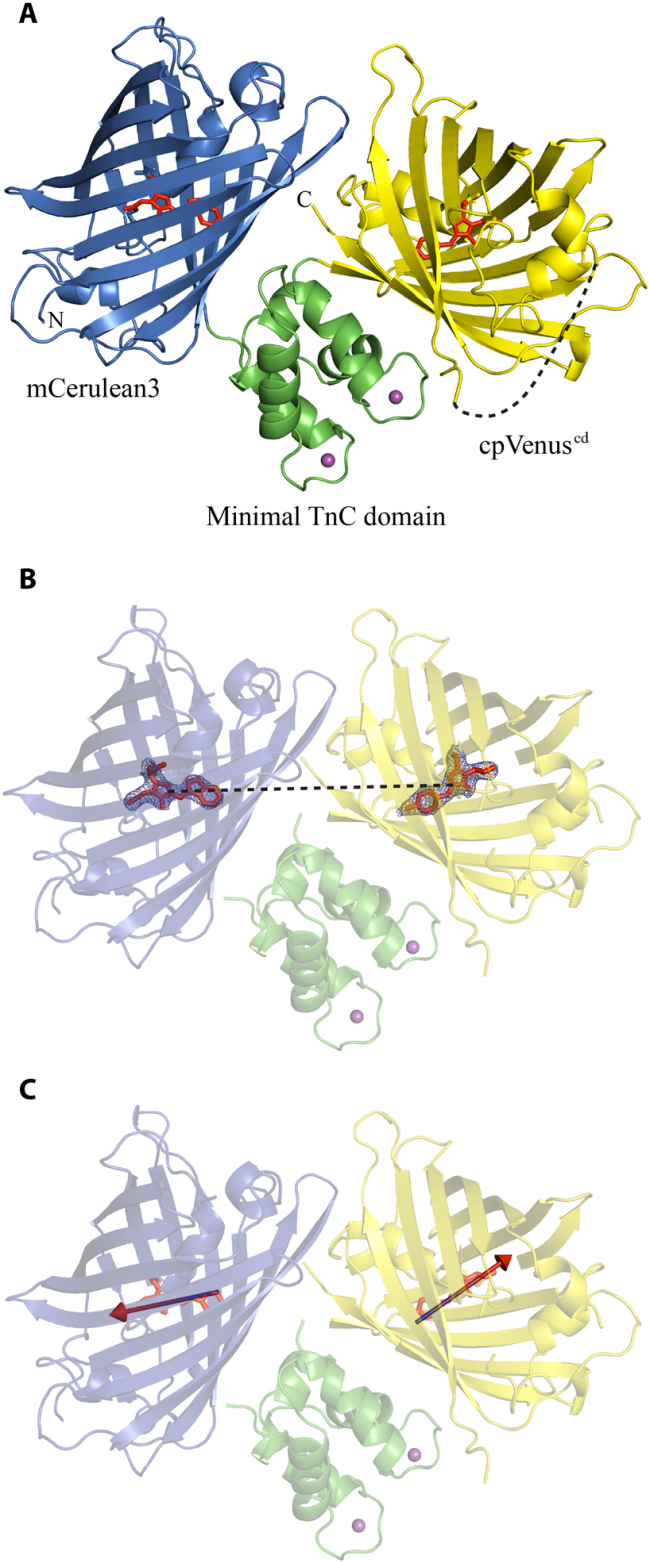
Cartoon representation of the x-ray structure of Twitch-2B. (**A**) The fluorophores of mCerulean3 and cpVenus^cd^ are depicted as stick structures in red. The calcium ions of the minimal TnC domain are shown as magenta spheres. The disordered linker connecting the original N and C termini of Venus is shown as a dashed line. (**B**) The distance between the centers of mass of the two fluorophores of Twitch-2B is depicted as a dashed line. (**C**) The transition dipole moments of the two fluorophores of Twitch-2B as obtained from (*15*) are depicted as arrows.

Numerous genetically encoded Förster resonance energy transfer (FRET) biosensors have been generated to study signaling inside living cells (*1*). In the prototypical Cameleons, calmodulin and its binding peptide M13 are sandwiched between Cyan and Yellow Fluorescent Proteins (*2*). Calmodulin wraps around M13 upon calcium binding, thereby changing the distance and orientation between the two fluorophores, resulting in FRET enhancement. Engineering of linker amino acids between the fluorescent proteins and calmodulin was shown to be crucial in optimizing calcium-induced FRET (*3*). The recently developed FRET-based calcium sensor Twitch-2B uses a minimal calcium-binding moiety derived from the C-terminal globular domain of toadfish Troponin C (TnC) fused between the mCerulean3 donor (*4*) and a circular permutation of the yellow fluorescent protein Venus (cpVenus^cd^) (*5*, *6*), which acts as the acceptor. Large-scale diversification and screening, predominantly of the residues linking the minimal calcium-binding domain and the fluorescent proteins, has been used to optimize FRET changes in this indicator (*7*, *8*). So far, only low-resolution structural data are available for any FRET biosensor (*8*–*10*). Here, we present the crystal structure of Twitch-2B (Fig. 1A) (*8*). It depicts the calcium-bound state and is, to our knowledge, the first crystal structure of any fluorescent protein FRET biosensor. We find that the structure-based prediction of FRET efficiency in Twitch-2B is significantly higher than the experimental efficiency. We address this issue with advanced nuclear magnetic resonance (NMR) methods and show that intrinsic dynamics is the reason for this discrepancy. Putting all information together allows us to design a rigidified mutant of Twitch-2B with increased FRET efficiency in solution.

## RESULTS AND DISCUSSION

### Crystal structure of Twitch-2B

**Fig. 2:**
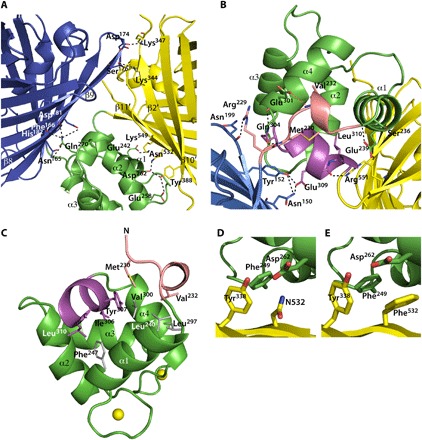
Structural details of Twitch-2B. (**A**) Polar interactions between residues of the minimal TnC domain, mCerulean3, and cpVenus^cd^ are depicted as dashed lines. The residues are shown as sticks. (**B**) Polar interactions mediated by residues (shown as sticks) from the linkers between mCerulean3 and the calcium-binding domain (in salmon), as well as interactions between cpVenus^cd^ and the calcium-binding domain (in magenta). (**C**) Hydrophobic interactions between residues (shown as sticks) from the linkers between mCerulean3 and the calcium-binding domain (in salmon), as well as the linker between cpVenus^cd^ and the calcium-binding domain (in magenta) with residues (in gray) from the core of the minimal TnC domain. (**D** and **E**) Close-up views of the region around N532 of Twitch-2B and of the N532F mutant of Twitch-2B (Twitch-6).

We solved the structure at a resolution of 2.5 Å (table S1A). There are two monomers in the asymmetric unit (fig. S1A). They present identical conformations of the individual domains [root mean square deviations (RMSDs) below 0.2 Å] and a slightly different interdomain conformation (RMSD of 0.992 Å) while apparently not assembling as a symmetric homodimer. Their interface (fig. S1B), covering 630 Å^2^ (*11*), is actually considerably smaller than a stable dimer interface (*12*). In addition, small-angle x-ray scattering (SAXS) data show that, in solution, Twitch-2B is monomeric (fig. S2). Therefore, we focus our description here on monomer A. The crystal structure reveals the positioning of the donor and the acceptor relative to the minimal calcium-binding TnC domain as well as the structure of the optimized linkers (Fig. 1). The structure of the calcium-binding domain is highly similar to the C-terminal globular domain of chicken TnC (RMSD of 0.84 Å) (*13*), to the NMR structure solved previously (*8*), and to that of calmodulin (RMSD of 1.08 Å) (*14*). The major axes of the two fluorescent protein β barrel domains are oriented at a nearly perpendicular angle to each other. The β barrels barely make contact with each other (Fig. 2A), with a very small common interface (150 Å^2^). The interfaces of the minimal calcium-binding domain with mCerulean3 and cpVenus^cd^ are also relatively small, covering only 257 and 351 Å^2^, respectively (see below). The interactions are mostly of hydrophilic character (Fig. 2A).

The linker between mCerulean3 and the calcium-binding domain (V_232_ADA) forms a 3_10_ helix, which is tightly held in place by backbone hydrogen bonds from V232 and S236 in mCerulean3 to E301 and E239 of the calcium-binding domain (Fig. 2B). Next, the linker between the calcium-binding domain and cpVenus^cd^ (P_305_IYPEL) forms one and a half α-helical turns (Fig. 2, B and C), the backbone carbonyls of E309 and L310 forming hydrogen bonds to the side chain of R551 (Fig. 2B) of cpVenus^cd^. The side chain of E309 also forms a hydrogen bond to Y152 of mCerulean3, tightly knitting the three domains together. Residues I306, Y307, and L310 of this short helix take part in a network of hydrophobic contacts (Fig. 2C). Obviously, the screening for optimal linkers (*8*) resulted in sequences with helical features integrating very well into the structure of the minimal TnC domain while these linkers hold in place the donor and acceptor domain mostly by polar interactions.

### Calculation of structure-based FRET efficiency

The structure provided crucial information for FRET calculations. First, the distance between the centers of mass of the fluorophores is 3.65 nm (Fig. 1B). Next, within the structure, the fluorophores of mCerulean3 and cpVenus^cd^ are aligned in a head-to-head configuration. Thus, we could exactly determine the relative orientation of the dipole moments of the fluorophores (Fig. 1C; see Materials and Methods) that are available from density functional theory calculations (*15*). With this combined information, we calculated an orientation factor, κ^2^, of 1.98 (Eq. 1; Materials and Methods) and a Förster distance, *R*_0_, of 6.9 nm for the mCerulean3/cpVenus^cd^ FRET pair (Eq. 3; Materials and Methods). With these parameters, using the Förster equation, $E=R_{0}^{6}/(R_{0}^{6}+r^{6})$, the theoretical FRET efficiency, *E*, of Twitch-2B was determined to be 0.98. The FRET efficiency experimentally determined by donor dequenching is 0.78 (fig. S3A), significantly lower than that derived from the crystal structure.

The two Twitch-2B monomers in the asymmetric unit (fig. S1A) not only have a very similar conformation (backbone RMSD of 0.992 Å) but also make very similar crystal packing contacts (fig. S1C). Therefore, we conclude that the orientation of the domains in the monomer is not per se constrained by the crystal packing but mostly by the intramonomer interactions described above (Fig. 2A) and is very likely selected from a pool of preexisting conformations in solution. As the interdomain interfaces in the Twitch-2B monomer are relatively small (Fig. 2A), high flexibility in solution might be the reason for the observed decrease in FRET efficiency. To investigate this hypothesis, we turned our attention to advanced NMR methods next.

### NMR investigation of biosensor dynamics

To gain insight into possible dynamics, we used paramagnetic NMR (*16*) with a Twitch-2B sample where the two TnC calcium-binding sites were loaded with dysprosium (Dy). The anisotropic magnetic susceptibility of the TnC-Dy_2_ complex induces a paramagnetic alignment tensor that can be determined from the structure (*17*) (see Materials and Methods). We used 900-MHz and 1.1-GHz spectrometers since the alignment tensor depends on the magnetic field quadratically. If a given fluorescent protein is rigid with respect to TnC, then the alignment tensor that it experiences is identical to that of TnC. If, however, the fluorescent protein is dynamic with respect to the TnC, then this motion will reduce the alignment tensor of the former (*16*, *18*). This allows quantifying the dynamics of the fluorescent proteins with respect to TnC. While dipolar couplings average out in isotropic solution, due to random isotropic tumbling, the paramagnetically induced alignment tensors lead to an anisotropic orientation distribution of TnC and, subsequently, of the attached green fluorescent proteins in solution, which results in the incomplete averaging of the dipolar couplings, allowing the observation of residual dipolar couplings (RDCs). We determined the RDCs of the methyl groups of the paramagnetically aligned Twitch-2B (*19*) (see Materials and Methods). The range of RDCs observed is sufficient to measure the size of the alignment tensor (*20*) such that assignment of the methyl groups was not necessary.

**Fig. 3:**
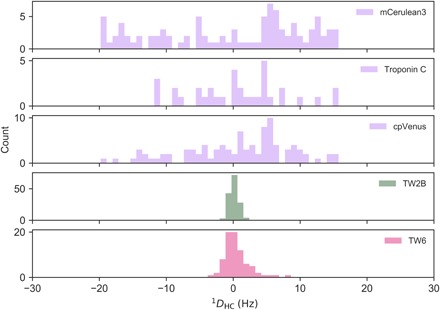
Histograms of the paramagnetic RDC data. Predictions of RDC of methyl groups in the two fluorescent protein domains and TnC using the x-ray structure (in purple). The alignment tensor induced by the two dysprosium ions bound to TnC results from the translation of the tensor derived from calmodulin (see Materials and Methods). Experimental RDCs from paramagnetic NMR of Twitch-2B (in green) and Twitch-6 (in magenta). The range is reduced by a factor of 10 and 5 for Twitch-2B and Twitch-6, respectively.

We found the range of RDC values and thus the alignment tensor experienced by the fluorescent proteins to be 10 times smaller than those calculated from the rigid x-ray structure (Fig. 3; see Materials and Methods). Thus, dynamics must be the reason behind the mismatch between the calculated and experimental FRET efficiencies. The crystal structure can only be part of a dynamic conformational ensemble in solution.

### Structure-based design of mutant with improved FRET efficiency

Assuming structural integrity of the single domains, we hypothesized that the linker regions are the pivot of those dynamics. The interfaces between the TnC domain and the donor and acceptor domains are dominated by polar interactions (Fig. 2A). We speculated that replacing those interactions by hydrophobic contacts would rigidify the linkers and increase the experimental FRET efficiency. Toward this goal, we designed the mutation N532F (Fig. 2, D and E) on the surface of cpVenus^cd^, creating a new interaction with F249 of the calcium-binding domain (Fig. 2D). As expected, this mutation effected a substantial increase in the maximal FRET ratio change from 800 to 1100% in vitro (fig. S4). In the crystal structure of this mutant (Twitch-6; table S2), the side chain of F532 indeed binds into a hydrophobic pocket formed by the side chains of F249, Asp262, and Y338 (Fig. 2E). Otherwise, the structures of Twitch-6 and Twitch-2B are very similar (RMSD of 0.25 Å), resulting in nearly identical theoretical FRET efficiencies (see the Supplementary Materials). With this design, the experimental FRET efficiency of Twitch-6 increased to 0.90, from 0.78 for Twitch-2B (fig. S3B), and the range of RDCs measured from Twitch-6 doubled from that of Twitch-2B (Fig. 3). This indicates the tightening of the interface between the TnC domain and cpVenus^cd^, and thus the reduction of the dynamics between the domains, is the reason for the increased FRET.

### Conformational ensembles in solution

**Fig. 4:**
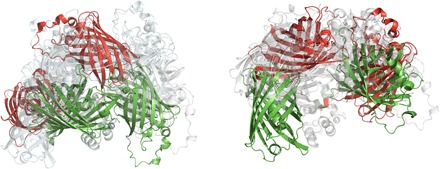
Ensembles of Twitch proteins consistent with the measured RDCs and FRET efficiencies. Ensembles for Twitch-2B (left) and Twitch-6 (right) contain six structures each and the largest deviating structures are shown in green and red. States that are not these extreme conformations are transparent.

Having identified dynamics as the reason for the reduced FRET efficiency of Twitch-2B in solution, we wanted to gain insight into the conformational space resulting from this dynamics. For this purpose, we selected conformational ensembles exploring backbone dynamics solely on the dynamic linker regions between the fluorescent protein domains and the TnC domain (see Materials and Methods) by scoring over 1 million six-membered ensembles against the observed RDCs and FRET efficiency (Table 1, Fig. 4, and Materials and Methods). Among all possible ensembles, we selected the one best reproducing both the experimental FRET efficiency and the range of RDCs (Table 1). This ensemble fully explains how flexibility in the disordered residues of the linker regions results in the observed reduction of FRET efficiency and RDC range.

**Table 1:** Ensemble selection results.

	**Experimental**		Ensemble*		
	**RDC^†^**	**FRET**	**RDC^†^**	**FRET**	**Q factor^‡^**
Twitch-2B	4.29	0.78	5.60	0.79	0.31
Twitch-6	11.43	0.90	9.29	0.88	0.07

*Results from the best-fitting ensembles of six structures selected with the protocol in Materials and Methods.

†RDCs shown are the distribution ranges of the experimental and ensemble parameters. The experimental (ensemble) RDC ranges from −2.01 to 2.27 Hz (−2.50 to +3.10 Hz) for Twitch-2B and from −3.48 to +7.95 Hz (−2.64 to +6.65 Hz) for Twitch-6.

‡An aggregated RMS score of RDC and FRET data (see Materials and Methods).

### Implications for improved FRET sensor design

In summary, we obtained the crystal structure of the fluorescent calcium biosensor Twitch-2B, and together with the dynamics of the linkers determined from paramagnetic NMR, we quantified the FRET efficiency. As dynamics was limiting the FRET efficiency, we successfully designed a rigidified mutant with increased FRET. Thus, structural and dynamic characterization of ratiometric FRET sensors provided design principles that might be applicable to other systems where FRET effects are used to sense signals.

## MATERIALS AND METHODS

### Cloning, expression, and purification of Twitch-2B and Twitch-6

The Twitch-2B construct has been described before (*8*). For the present study, the coding sequence of the three-domain fusion protein was cloned into a modified pET16b vector coding for a fusion protein with N-terminal His_7_-tag and tabac etch virus (TEV) recognition cleavage sequence. The Twitch-2B N532F mutant (Twitch-6) was generated using the QuikChange site-directed mutagenesis kit (Agilent). The pET16bTEV-Twitch-2B and pET16bTEV-Twitch-6 expression constructs were transformed into *Escherichia coli* strain BL21 (DE3). Protein expression was carried out at 303 K by induction with 0.5 mM isopropyl β-d-1-thiogalactopyranoside. The cells were harvested 7 hours after induction. Selenomethionine-labeled Twitch-2B protein was overexpressed in the methionine-auxotroph *E. coli* strain B834 in minimal medium supplemented with (+)-l-selenomethionine according to the EMBL (European Molecular Biology Laboratory) protein-expression group (www.embl.de).

The cell pellet from 1 liter of shaking culture was resuspended in 60 ml of lysis buffer [20 mM tris-HCl (pH 7.9), 300 mM NaCl, 20 mM imidazole, 0.5 mM phenylmethylsulfonyl fluoride, with one tablet of cOmplete EDTA-free inhibitors (Roche) per 100 ml of lysis buffer]. The cells were lysed by sonication followed by centrifugation at 27,000*g* and 277 K. From the supernatant, the recombinant protein was purified by immobilized metal-affinity chromatography on 3 ml of Ni–nitrilotriacetic acid (NTA) agarose resin (Qiagen). The His_7_-fusion tag was cleaved off with TEV protease and removed by incubation with 1 ml of Ni-NTA agarose resin. The protein was dialyzed against 20 mM tris (pH 7.0) and 150 mM NaCl. After adjusting the ammonium sulfate concentration in the protein solution to 1 M, the protein was further purified by hydrophobic interaction chromatography on a 10-ml Phenyl Sepharose (GE Healthcare) column. The protein was eluted from this column with a 50-ml gradient from 1 to 0 M ammonium sulfate. Fractions containing the protein were pooled and concentrated to a volume of 2.5 ml with a 30-kDa MWCO (molecular weight cut-off) ultrafiltration concentrator (Vivascience). Last, the protein was purified by size exclusion chromatography on a HiLoad 26/60 Superdex 200-pg gel filtration column. The peak fractions were pooled, dialyzed against 20 mM tris-HCl (pH 7.0), 100 mM NaCl, and 5 mM CaCl_2_, and the protein concentration was adjusted to 20 mg/ml.

### Fluorescence spectroscopy

For in vitro spectroscopy of recombinant Twitch-2B, the protein was purified from *E. coli* using Ni-NTA resin, as described (*6*). Spectroscopy was performed on a Cary Eclipse Spectrophotometer (Varian). Donor dequenching of Twitch-2B was performed by digesting Twitch-2B in a calcium-bound state overnight at room temperature with chymotrypsin (70 U/ml; Sigma-Aldrich) while recording FRET. A small remaining cpVenus^cd^ emission after chymotrypsin digestion was selectively photobleached (5 min) with an array of six Luxeon Lumiled light-emitting diodes peaking at 530 nm, for a total power dissipation of 14.7 W and 870 lumen. A 500-nm LP (long-pass) filter was used to protect mCerulean3 from bleaching. Calcium-bound Twitch-6 was very resistant to protease digestion. Therefore, EGTA (final concentration, 5 mM) was added during chymotrypsin digestion to obtain the spectrum of dequenched mCerulean3.

### Crystallization, data collection, and structure determination

Crystals of Twitch-2B and Twitch-6 were obtained by vapor diffusion mixing of 1 μl of protein solution with 1 μl of well solution [0.2 M Na-formiate (pH 7.0), 5 mM CaCl_2_, and 18 to 20% polyethylene glycol 3350]. Crystals were cryoprotected by transferring them to a well solution supplemented with 16 to 18% glycerol for 1 min and flash-cooled by plunging them into liquid nitrogen.

Data collection was performed at PXII, SLS, Switzerland, using a PILATUS 6M detector (Dectris). Native data were collected at 100 K at a wavelength of 1 Å. Selenomethionine derivative data were measured at 0.98 Å. All data were processed with x-ray detector software (XDS) (*21*) and scaled with SADABS (Bruker AXS). Space group determination and statistical analysis were carried out using XPREP (Bruker AXS). Phasing was performed with AutoSol (*22*).

The initial model was built with AutoBuild and refined twice with phenix.refine (*23*) with intermediate manual model building with Coot (*24*). The final model was obtained by combined manual tracing (Coot) and refinement using Refmac5 (*25*). In the Ramachandran plot, 96.69% of the residues were located in the favored region, 2.72% in the allowed region, and 0.58% of the residues were outliers. The crystal structure of Twitch-6 was solved with PHASER (*26*) using PDB (Protein Data Bank) entry 6GEL as the search model. Model building and refinement were performed as described for Twitch-2B. For this mutant, 96.68% of the residues fell into the favored region of the Ramachandran plot, 2.83% fell into the allowed region, and 0.49% were outliers.

### FRET calculations

The orientation factor, κ^2^, can be extracted from structural information as follows

$$\kappa^{2}={\left(\text{cos}\;\theta_{T}\;-\;3\;\text{cos}\;\theta_{D}\;\text{cos}\;\theta_{A}\right)}^{2}$$ (1)

where θ_T_ is the angle between the emission transition dipole of the donor and the absorption transition dipole of the acceptor; θ_D_ and θ_A_ are the angles between these dipoles and the vector, *r*, joining the donor and the acceptor fluorophores (*27*). The orientation of the transition dipole moments relative to the *C* → *O* bond vector (ω) in degrees

$$\omega_{D}=73{}^{\circ}$$

$$\omega_{A}=76{}^{\circ}$$

were taken from Ansbacher *et al.* (*15*), and the angular parameters were extracted from the crystallographic coordinates, in angular units

$$\theta_{T}=152.95{}^{\circ}$$

$$\theta_{D}=149.17{}^{\circ}$$

$$\theta_{A}=26.79{}^{\circ}$$

The detailed calculations are explained in data file S1. The overlap integral, *J*(λ), was calculated from the experimental absorbance and emission spectra of the isolated cpVenus and mCerulean3 domains, respectively (fig. S5), with a Python script included as supplementary information. The *J*(λ) was determined to be 2.052 × 10^15^ M^−1^ cm^−1^ nm^4^, as follows

$$J(\lambda )=\int_{0}^{\infty }F_{D}(\lambda )\varepsilon_{A}(\lambda )\lambda^{4}d\lambda =\frac{\int_{0}^{\infty }F_{D}(\lambda )\varepsilon_{A}(\lambda )\lambda^{4}d\lambda }{\int_{0}^{\infty }F_{D}(\lambda )d\lambda }$$ (2)

where *F*_D_(λ) is the normalized fluorescence intensity of the donor in the wavelength range λ to λ + Δλ. ε_A_(λ) is the extinction coefficient of the acceptor at λ.

The Förster distance, *R*_0_, can be calculated from the previously derived experimental parameters

$$R_{0}=0.211{\left(\kappa^{2}n^{-4}Q_{D}J(\lambda )\right)}^{1/6}$$ (3)

where *Q*_D_ (0.87) is the quantum yield of the donor in the absence of acceptor (*4*) and *n*, (1.33) the refractive index of the aqueous medium.

Last, the efficiency of energy transfer, *E*, can be calculated as the ratio of the transfer rate to the total decay rate of the donor in the presence of acceptor

$$E=\frac{R_{0}^{6}}{R_{0}^{6}+r^{6}}$$ (4)

Following this procedure, the FRET efficiency was determined to be *E* = 0.979, from the crystallographic structure of Twitch-2B. The equivalent calculation performed with the structure of the mutant Twitch-6 gives an *E* = 0.983 (see data files S1 and S2).

### NMR spectroscopy

We expressed the proteins Twitch-2B and Twitch-6 in Toronto minimal medium prepared with 100% D_2_O and perdeuterated d-glucose and supplemented with the amino acid precursors α-ketobutyric acid (methyl-13C, 3,3-D2) and α-ketoisovaleric acid (3-methyl-13C, 3,4,4,4-D4), thus selectively labeling with ^13^C and ^1^H atoms only of the methyl groups of residues valine, leucine, and isoleucine while keeping the rest of the C atoms as ^12^C and protons as ^2^H (*19*).

First, the isotropic sample spectra were acquired in buffer A [20 mM Mops (pH 7.0), 100 mM NaCl, and 5 mM CaCl_2_ in 100% D_2_O]. Next, the proteins were dialyzed against buffer B [20 mM Mops (pH 7.0), 100 mM NaCl, and 10 mM EDTA] followed by dialysis against buffer C [20 mM Mops (pH 7.0) and 100 mM NaCl] and finally exchanged to buffer C prepared in 100% D_2_O containing three equivalents of dysprosium before NMR measurements. The protein concentration in the samples was approximately 0.5 mM.

The samples were tested with methyl-TROSY experiments (*19*, *28*) (fig. S7) at 900 MHz and 1.1 GHz, and *J* couplings and J + RDC were determined using a *J*-modulated methyl-TROSY (*29*) experiment depicted in fig. S6 as matrices of 2048 × 128 complex data points with 96 transients per (*t*_1_) increment. The total *J*-modulation delays used were as follows: 4, 6, 8, 10, 12, 14, 16, 18, and 20 ms (fig. S8). NMR experiments were acquired using a 5-mm TCI (triple resonance cryoprobe with Inverse detection) on a 900-MHz spectrometer and a 3-mm TCI cryoprobe on a 1.1-GHz spectrometer, both equipped with NEO consoles (Bruker). The intensities (maximum amplitude) of the signals were extracted using CARA (computer aided resonance assignment) (*30*) on NMRPipe-processed experiments (*31*) and analyzed with Python scripts (fig. S8) following Pederson *et al.* (*29*).

### Calculation of the paramagnetic tensor

We took the paramagnetic tensor from the calmodulin-IQ complex bound to dysprosium from (*18*) and calculated the total tensor of the doubly occupied TnC calcium-binding domain. The TnC calcium-binding site 1 overlaps with the lanthanide-binding site of calmodulin (CaM N60D). We rotated the alignment matrix from the calcium-binding site from CaM onto the second calcium-binding site of TnC and added it up to the calcium-binding site 1 tensor, thus obtaining the total TnC tensor. This tensor was then used to calculate the RDCs from the paramagnetic Twitch proteins

$$A^{\text{CaM}}=(\begin{array}{ccc} 1.05\;10^{-31} & -1.92\;10^{-32} & 2.04\;10^{-31}\\ -1.92\;10^{-32} & -1.22\;10^{-31} & 6.46\;10^{-32}\\ 2.04\;10^{-31} & 6.46\;10^{-32} & 1.67\;10^{-32} \end{array})$$

$$A^{\text{Twitch}}=(\begin{array}{ccc} 1.46\;10^{-31} & 8.39\;10^{-32} & 3.46\;10^{-31}\\ 8.39\;10^{-32} & -1.06\;10^{-31} & 2.26\;10^{-32}\\ 3.46\;10^{-31} & 2.26\;10^{-32} & -3.92\;10^{-32} \end{array})$$

The tensors are given in m^3^ M^−1^.

### Ensemble generation

First, we individually modeled all possible backbone dihedral angles (ϕ and ψ; sampling in steps of 60°) of the linker residues (Arg^229^ to Gln^231^ for mCerulean3 and Met^311^ to Gly^313^ for cpVenus), which did not result in a steric clash between one of the modified fluorescent protein domains and the TnC domain. Each of the two linker regions was modeled independently. Of all possible ϕ, ψ combinations (117,649), the rotation of mCerulean3 resulted in 477 possible conformations, while cpVenus allowed 84 conformations, providing altogether 40,086 possible conformations.

Second, we combined the possible structures for mCerulean-TnC and TnC-cpVenus at random in ensembles of six members. We sampled randomly 1 million six-membered ensembles out of the 40,068 possible ϕ, ψ combinations of the linkers between mCerulean and TnC and between TnC and cpVenus to ensure a proper exploration of the conformational space of Twitch.

Third, we calculated the RDC range (using the tensor derived as described above) and FRET of the ensembles and compared them with the experimental values by defining an ensemble quality factor

$$Q_{\text{ens}}=\sum_{i=1}^{i=6}\sqrt{{\left(\frac{{\text{RDC}}_{i}-{\text{RDC}}_{e}}{{\text{RDC}}_{e}}\right)}^{2}+{\left(\frac{{\text{FRET}}_{i}-{\text{FRET}}_{e}}{{\text{FRET}}_{e}}\right)}^{2}}$$

where RDC*_i_* are distribution ranges, the subindex *e* indicates an experimental value, and *i* indicates the value from an ensemble member. Such quality factor value is different from 0 if the agreements of the predicted RDC and FRET responses from the ensemble deviate from the experimental data and it is 0 in the case of complete agreement. Last, the ensembles were sorted by their *Q*_ens_, and the lowest one was selected.

### SAXS measurements

SAXS data were collected at beamline BM29 of the European Synchrotron Radiation Facility (ESRF) in Grenoble, France, using the automatic sample changer (*32*). The protein was dialyzed either against buffer A [20 mM Mops (pH 7.0), 100 mM NaCl, and 5 mM CaCl_2_] (calcium-bound state) or against buffer B [20 mM Mops (pH 7.0), 100 mM NaCl, and 10 mM EDTA] (calcium-free state). Before measurement, the protein was centrifuged to remove any larger particles. Samples were measured at concentrations of 2.5, 10, and 20 mg/ml. The dialysis buffer was used for reference buffer correction. Data were collected at 293 K using a wavelength of 0.995 Å and a sample-to-detector distance of 2.867 m. One hundred microliters was loaded of each sample concentration, and 10 frames of were collected and merged. Samples were flowed constantly into the cell to minimize radiation damage effects. The detector images were integrated and reduced to one-dimensional scattering curves, and buffer contributions to scattering were subtracted using the beamline software BsxCuBE. Further data processing was performed automatically using the online EDNA pipeline (*33*) to assess sample quality and radiation damage effects. No protein aggregation or radiation damage was observed.

Data were further analyzed with the ATSAS software package (*34*). Briefly, primary data reduction and analysis were performed using the programs PRIMUS (*35*) and GNOM (*36*).

The theoretical scattering from the crystal structure was calculated using the program CRYSOL (*37*) (fig. S2), and the molecular weights were calculated using a bovine serum albumin sample as the standard.

## Supplementary Material

http://advances.sciencemag.org/cgi/content/full/5/8/eaaw4988/DC1

Download PDF

Data file S1

Data file S2

## SUPPLEMENTARY MATERIALS

Supplementary material for this article is available at http://advances.sciencemag.org/cgi/content/full/5/8/eaaw4988/DC1 Table S1. Data collection and refinement statistics for Twitch-2B. Table S2. Data collection and refinement statistics for Twitch-6. Fig. S1. Crystal packing of Twitch-2B. Fig. S2. The SAXS data of Twitch-2B show monomeric state in solution. Fig. S3. Donor dequenching of the calcium-bound Twitch proteins. Fig. S4. Emission spectrum (excitation, 432 nm) of Twitch-6 (Twitch-2B N532F) in calcium-free state (black trace) and at calcium saturation (red trace). Fig. S5. Experimental fluorescence absorbance and emission spectra of the isolated cpVenus and mCerulean3, respectively. Fig. S6. Pulse-sequence scheme for the *J*-modulated HMQC-TROSY experiment used to determine ^1^*J*_HC_ couplings (Ca-loaded samples) and the corresponding ^1^*D*_HC_ (Dy-loaded samples). Fig. S7. Characterization of dynamics in Twitch-2B and Twitch-6 by paramagnetic NMR. Fig. S8. Example of intensity peak modulation of the *J*-modulated HMQC-TROSY experiment from Twitch-2B acquired at 1.1 GHz. Data file S1. Structure_Based_FRET_Twitch-2B.xlsx (Excel file). Data file S2. Structure_Based_FRET_Twitch-2B.py (Python script).
