# Supplementary material for: Dynamic tuning of FRET in a green fluorescent protein biosensor
Source: Sci Adv. 2019 Aug 7;5(8):eaaw4988. doi: 10.1126/sciadv.aaw4988 (PMC6685724; doi:10.1126/sciadv.aaw4988)
Supplement: http://advances.sciencemag.org/cgi/content/full/5/8/eaaw4988/DC1 [file supp_5_8_eaaw4988__index.html]

Science Advances | Science AdvancesAAASSearchScience AdvancesMenu

## Supplementary Materials

**The PDF file includes:**

- Table S1. Data collection and refinement statistics for Twitch-2B.
- Table S2. Data collection and refinement statistics for Twitch-6.
- Fig. S1. Crystal packing of Twitch-2B.
- Fig. S2. The SAXS data of Twitch-2B show monomeric state in solution.
- Fig. S3. Donor dequenching of the calcium-bound Twitch proteins.
- Fig. S4. Emission spectrum (excitation, 432 nm) of Twitch-6 (Twitch-2B N532F) in calcium-free state (black trace) and at calcium saturation (red trace).
- Fig. S5. Experimental fluorescence absorbance and emission spectra of the isolated cpVenus and mCerulean3, respectively.
- Fig. S6. Pulse-sequence scheme for the *J*-modulated HMQC-TROSY experiment used to determine 1*J*HC couplings (Ca-loaded samples) and the corresponding 1*D*HC (Dy-loaded samples).
- Fig. S7. Characterization of dynamics in Twitch-2B and Twitch-6 by paramagnetic NMR.
- Fig. S8. Example of intensity peak modulation of the *J*-modulated HMQC-TROSY experiment from Twitch-2B acquired at 1.1 GHz.

Download PDF

**Other Supplementary Material for this manuscript includes the following:**

- Data file S1. Structure\_Based\_FRET\_Twitch-2B.xlsx (Excel file).
- Data file S2. Structure\_Based\_FRET\_Twitch-2B.py (Python script).

**Files in this Data Supplement:**

- Adobe PDF - aaw4988\_SM.pdf
