# Supplementary material for: Dynamic tuning of FRET in a green fluorescent protein biosensor
Source: Sci Adv. 2019 Aug 7;5(8):eaaw4988. doi: 10.1126/sciadv.aaw4988 (PMC6685724; doi:10.1126/sciadv.aaw4988)
Supplement: Download PDF [file aaw4988_SM.pdf]

Science Advances
AAAS

advances.sciencemag.org/cgi/content/full/5/8/eaaw4988/DC1

Supplementary Materials for

Dynamic tuning of FRET in a green fluorescent protein biosensor

Pablo Trigo-Mourino, Thomas Thestrup, Oliver Griesbeck, Christian Griesinger, Stefan Becker*

*Corresponding author. Email: sbecker2@gwdg.de

Published 7 August 2019, *Sci. Adv.* **5**, eaaw4988 (2019)
DOI: 10.1126/sciadv.aaw4988

The PDF file includes:

Table S1. Data collection and refinement statistics for Twitch-2B.
Table S2. Data collection and refinement statistics for Twitch-6.
Fig. S1. Crystal packing of Twitch-2B.
Fig. S2. The SAXS data of Twitch-2B show monomeric state in solution.
Fig. S3. Donor dequenching of the calcium-bound Twitch proteins.
Fig. S4. Emission spectrum (excitation, 432 nm) of Twitch-6 (Twitch-2B N532F) in calcium-free state (black trace) and at calcium saturation (red trace).
Fig. S5. Experimental fluorescence absorbance and emission spectra of the isolated cpVenus and mCerulean3, respectively.
Fig. S6. Pulse-sequence scheme for the *J*-modulated HMQC-TROSY experiment used to determine $^{1}J_{HC}$ couplings (Ca-loaded samples) and the corresponding $^{1}D_{HC}$ (Dy-loaded samples).
Fig. S7. Characterization of dynamics in Twitch-2B and Twitch-6 by paramagnetic NMR.
Fig. S8. Example of intensity peak modulation of the *J*-modulated HMQC-TROSY experiment from Twitch-2B acquired at 1.1 GHz.

Other Supplementary Material for this manuscript includes the following:

(available at advances.sciencemag.org/cgi/content/full/5/8/eaaw4988/DC1)

Data file S1. Structure_Based_FRET_Twitch-2B.xlsx (Excel file).
Data file S2. Structure_Based_FRET_Twitch-2B.py (Python script).

Table S1. Data collection and refinement statistics for Twitch-2B.

	Native	Se-Met (Peak)
**Data collection**		
Space group	$P2_12_12_1$	
Cell dimensions		
a, *b*, *c* (Å)	58.08, 156.77, 169.28	58.26, 157.47, 169.42
α, β, γ (°)	90, 90, 90	90, 90, 90
Resolution (Å) *	2.51 (2.61-2.51)	2.71 (2.82-2.72)
R_{int} (%)	5.95 (38.61)	7.97 (38.54)
$I / \sigma I$	24.16 (3.32)	6.48 (5.73)
Completeness (%)	98.7 (95.7)	99.0 (91.3)
Redundancy	6.62 (6.31)	6.48 (5.73)
**Refinement**		
Resolution (Å)	2.51	
No. reflections	50452	
R_{work} / R_{free}	20.0/23.9	
No. atoms		
Protein		
Monomer A	4278	
Monomer B	4248	
Ca^{2+} ligand	4	
Water	113	
B-factors		
Protein		
Monomer A	63.2	
Monomer B	66.7	
Ca^{2+} ligand	65.3	
Water	45	
R.m.s. deviations		
Bond lengths (Å)	0.017	
Bond angles (°)	2.07	
Ramachandran plot (% of residues in regions)		
most favored	96.69	
additionally allowed	2.72	
disallowed	0.58	
PDB code	2GEL	

*Values in parentheses are for highest-resolution shell.

Table S2. Data collection and refinement statistics for Twitch-6.

	Native
**Data collection**	
Space group	$P2_12_12_1$
Cell dimensions	
a, b, c (Å)	57.62, 157.47, 169.42
α, β, γ (°)	90, 90, 90
Resolution (Å) *	2.47 (2.57-2.47)
R_{int} (%)	6.4 (52.44)
$I / \sigma I$	14.95 (1.97)
Completeness (%)	97.5 (80.9)
Redundancy	6.36 (5.0)
**Refinement**	
Resolution (Å)	2.47
No. reflections	51921
R_{work} / R_{free}	21.7/24.5
No. atoms	
Protein	
Monomer A	4927
Monomer B	4311
Ca^{2+} ligand	4
Water	78
B-factors	
Protein	
Monomer A	69.8
Monomer B	75.13
Ca^{2+} ligand	76
Water	55.17
R.m.s. deviations	
Bond lengths (Å)	0.02
Bond angles (°)	2.0
Ramachandran plot (% of residues in regions)	
most favored	96.68
additionally allowed	2.83
disallowed	0.49
PDB code	2GEZ

*Values in parentheses are for highest-resolution shell.

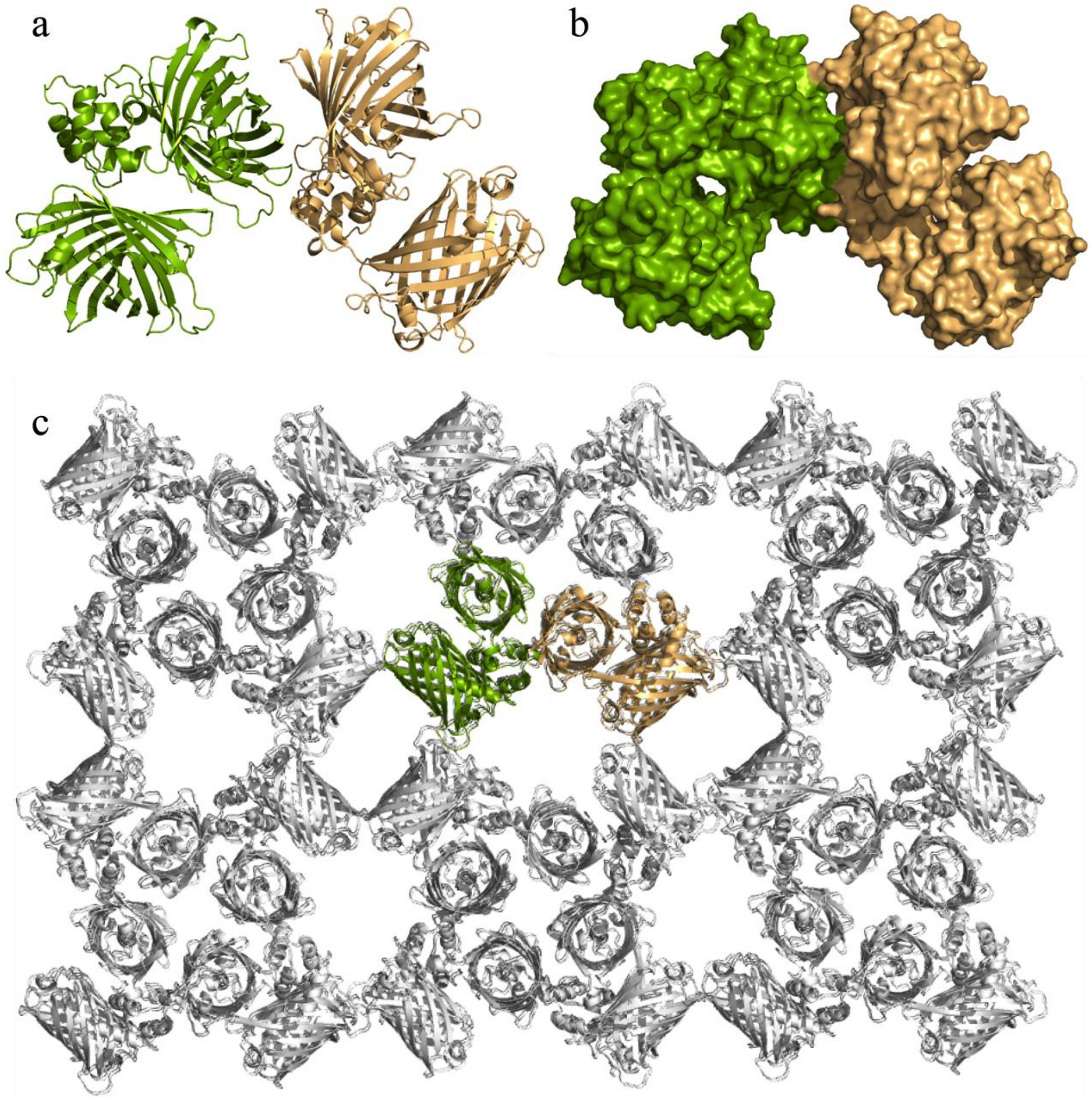

Fig. S1. Crystal packing of Twitch-2B. (a) There are 2 monomers in the asymmetric unit. (b) Their interface covers 630 Å^2. (c) Altogether, both monomers make very similar crystal contacts (view along cell axis A).

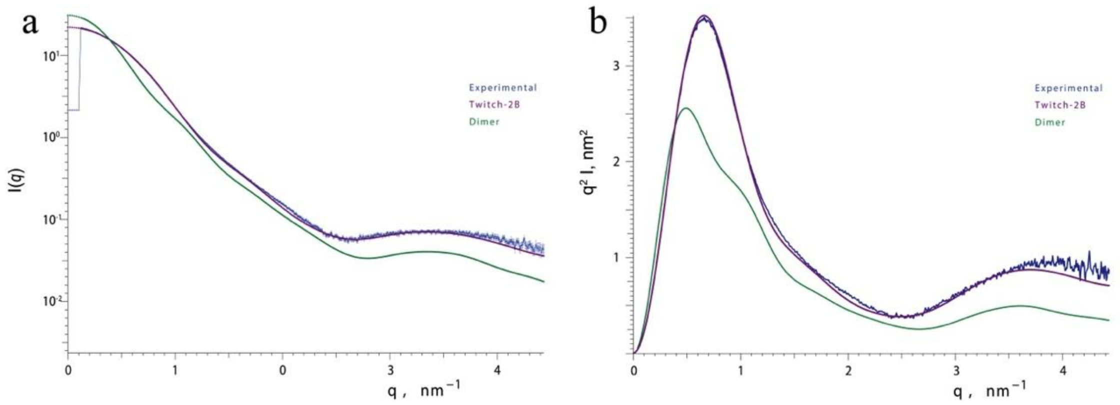

Fig. S2. The SAXS data of Twitch-2B show monomeric state in solution. (a) Experimental scattering curve (blue). X-ray structure based predictions for monomeric and dimeric state are shown in magenta and green, respectively. (b) The Kratky plot shows that the protein is clearly monomeric.

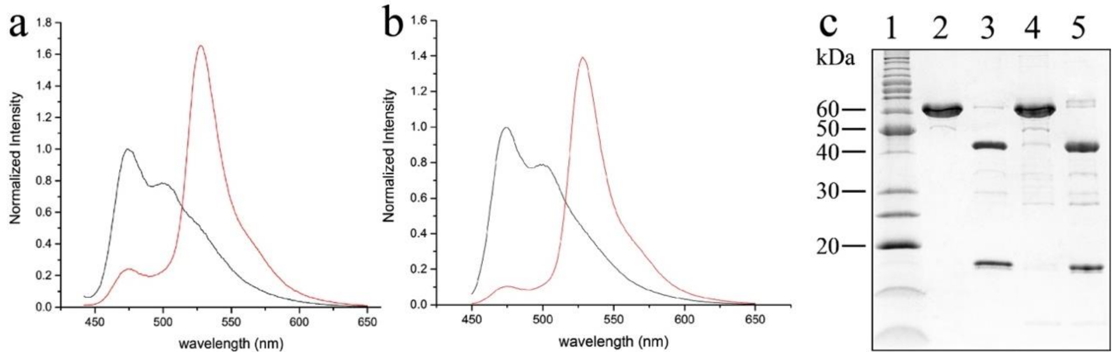

Fig. S3. Donor dequenching of the calcium-bound Twitch proteins. The emission spectra of the calcium-bound Twitch-2B (a) and Twitch-6 (b) (excitation 432 nm) were recorded before (red line) and after a 24h digest with chymotrypsin (70 units/ml) (black line). The digestion of Twitch-6 with chymotrypsin was performed in presence of 5 mM EGTA (see Materials and Methods). The spectra were normalized to the mCerulean3 emission maximum. In (c) the digestion of both proteins is visualized on a 15% SDS polyacrylamide gel stained with Coomassie R-250. Lane 1, protein size standards; lane 2, Twitch-2B undigested; lane 3, Twitch-2B digested with chymotrypsin; lane 4, Twitch-6 undigested; lane5, Twitch-6 digested with chymotrypsin. The masses of the digestion fragments were determined by electrospray mass spectrometry. The mass of the smaller digestion fragment is 20.218 kDa for Twitch-2B and 20.252 kDa for Twitch-6. The difference of 34 Da between the two masses is due to the N532F mutation in Twitch-6. Therefore the smaller digestion fragment could be clearly allocated to the C-terminal fragment starting at residue 376 of both proteins. For both Twitch-2B and Twitch-6 the mass of the larger digestion fragment was 42.368 kDa, comprising amino acids 1 to 375. Notably, this fragment contains the complete sequence of mCerulean3 that is obviously not affected by chymotrypsin digestion.

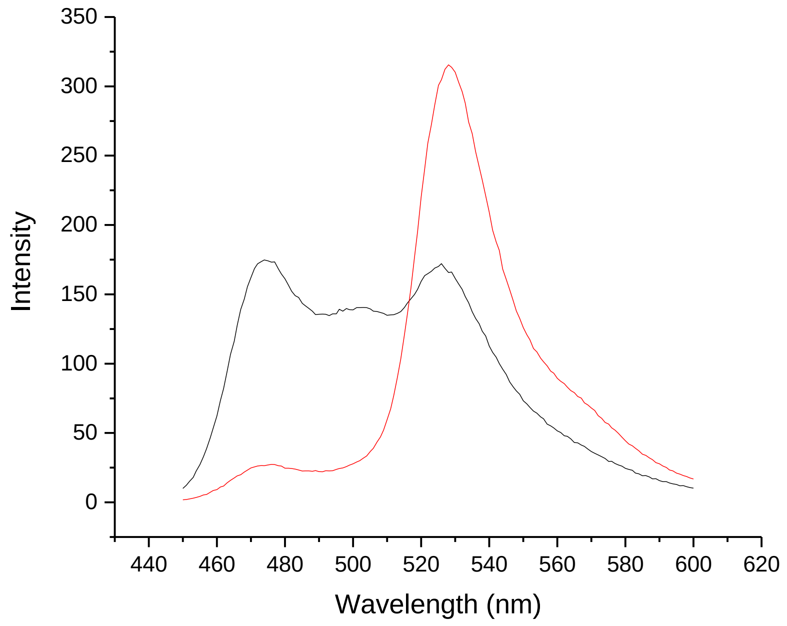

Fig. S4. Emission spectrum (excitation, 432 nm) of Twitch-6 (Twitch-2B N532F) in calcium-free state (black trace) and at calcium saturation (red trace). The overall ratio change was 1100%. Following our published protocol (*8*), the K_d for calcium affinity was determined to be 100 nM.

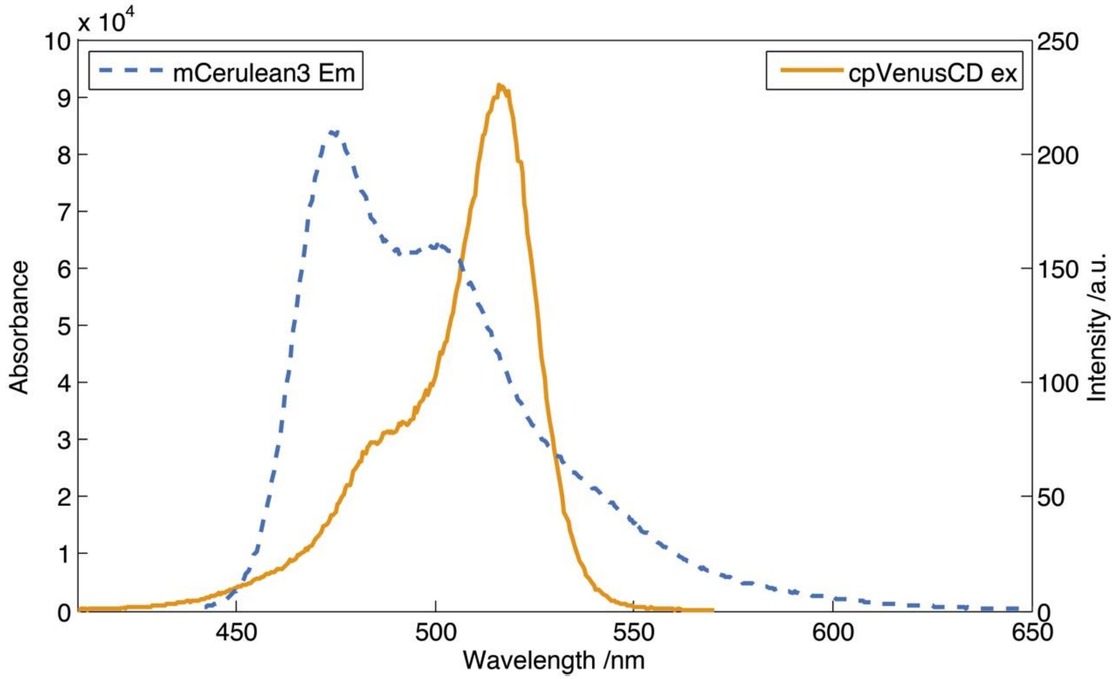

Fig. S5. Experimental fluorescence absorbance and emission spectra of the isolated cpVenus and mCerulean3, respectively. The overlap integral, $J(\lambda)$, calculated with Equation 2 as in ref. (*27*) is 2.052E+15 M^{-1} cm^{-1} nm^{4} (see Materials and Methods).

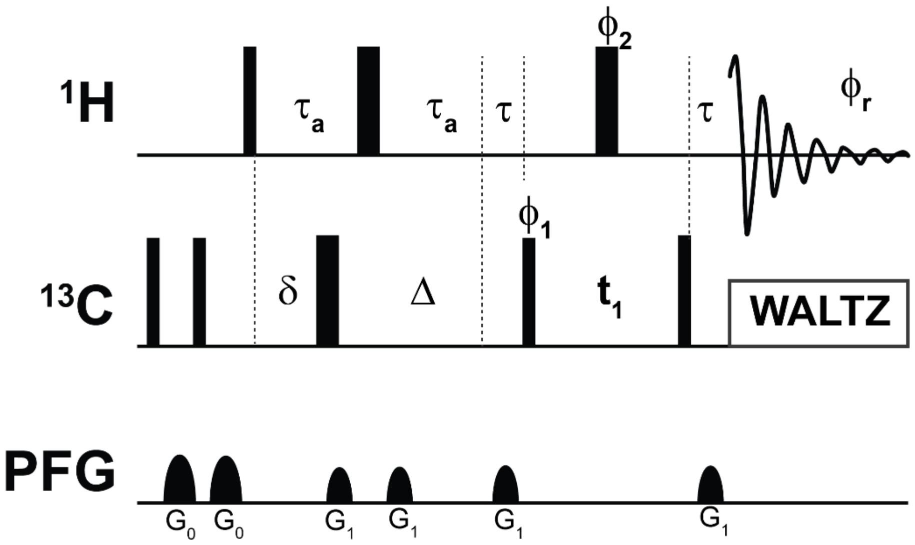

Fig. S6. Pulse-sequence scheme for the *J*-modulated HMQC-TROSY experiment used to determine $^1J_{HC}$ couplings (Ca-loaded samples) and the corresponding $^1D_{HC}$ (Dy-loaded samples) (29). The pulses have phase x unless indicated otherwise; ϕ_1: x, -x; ϕ_2: 2x, 2y, 2(-x), 2(-y); ϕ_r: x, -x; -x, x. Gradient pulses are set to 500 μs duration and have strengths of 50% and 30% of maximum strength for G_0 and G_1, respectively. The delay δ is equal to $\tau_a - \tau_{a'}$, and Δ to $\tau_a + \tau_{a'}$. Several of such delays are acquired to sample enough of the sine modulated intensity in order to fit for the coupling constant (see fig. S8). The delays used for this manuscript are: τ = 3.6 ms, τ_a = 10 ms; The J_{CH} modulation delays ($\tau + 2\tau_a - 22\tau_a'$) are: 4, 6, 8, 10, 12, 14, 16, 18 and 20 ms.

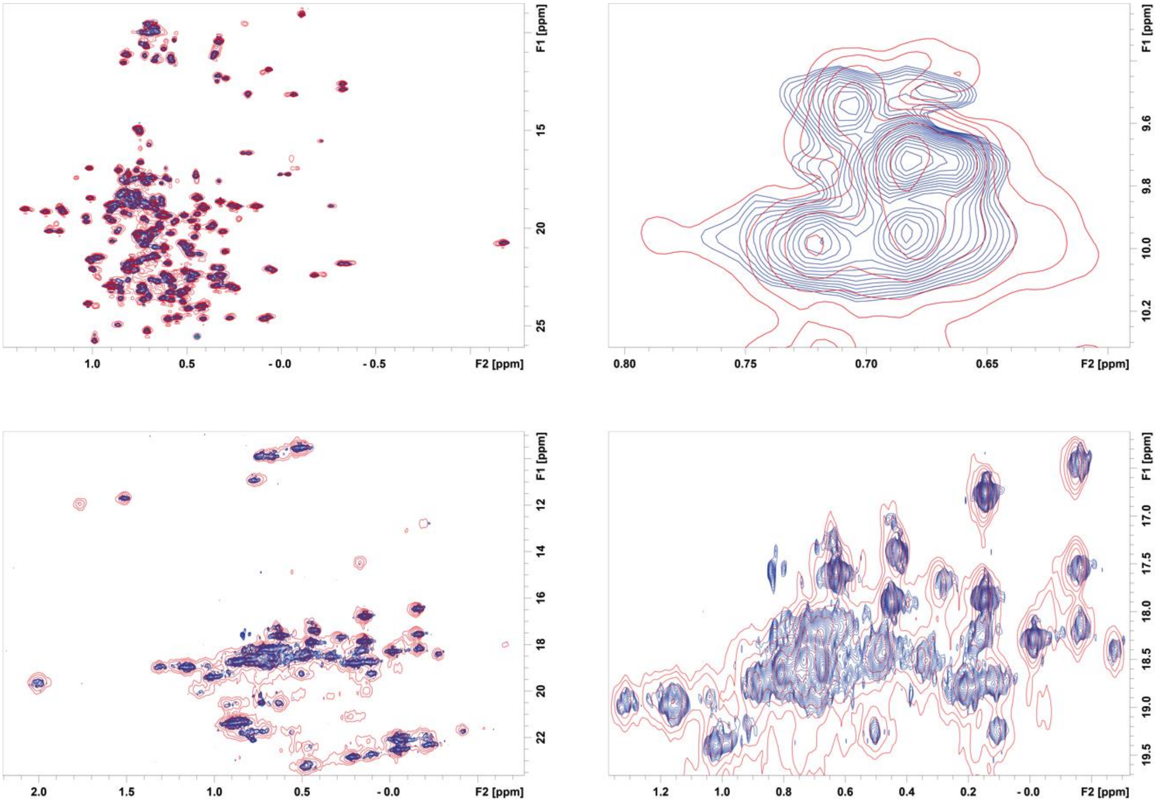

Fig. S7. Characterization of dynamics in Twitch-2B and Twitch-6 by paramagnetic NMR. HMQC-TROSY spectra overlay from Twitch-2B (top) and Twitch-6 (bottom) loaded with Dysprosium. Left, overview of the full methyl region of the HMQC; right, detailed view of a selected region. The overlay pictures spectra acquired at 900 MHz (red) and 1.1 GHz (blue), showing the enhanced spectral quality of the 1.1 GHz spectrometer. The striking difference in spectral quality between Twitch-2B and Twitch-6 is indicative of very different interdomain dynamics.

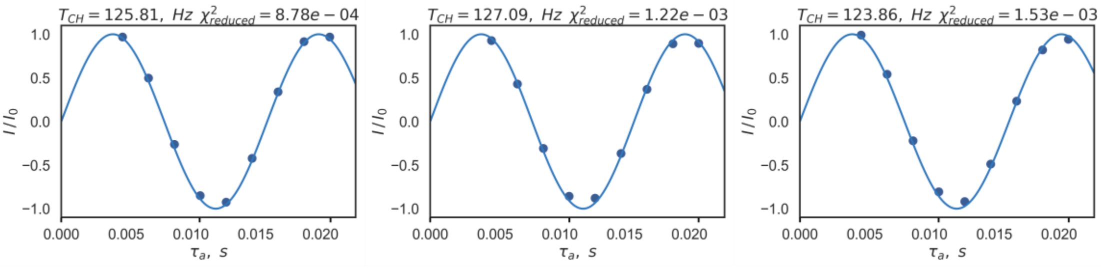

Fig. S8. Example of intensity peak modulation of the *J*-modulated HMQC-TROSY experiment from Twitch-2B acquired at 1.1 GHz. The maximum peak intensity (blue dots) is extracted as detailed in the Methods and fit to $\sin\left(\pi J_{CH}\left(2\delta+\tau\right)\right)=\sin\left(\pi J_{CH}\left(2\left(\tau_a-\tau_{a'}\right)+\tau\right)\right)$ in order to extract the coupling.
